# Investigation of known estimated glomerular filtration rate loci in patients with Type 2 diabetes

**DOI:** 10.1111/dme.12211

**Published:** 2013-05-14

**Authors:** H A Deshmukh, C N A Palmer, A D Morris, H M Colhoun

**Affiliations:** 1 Division of Population Health Sciences, University of DundeeDundee, UK; 2 Division of Cardiovascular and Diabetes Medicine, University of DundeeDundee, UK

## Abstract

**Aims:**

To replicate the association of genetic variants with estimated glomerular filtration rate (GFR) and albuminuria, which has been found in recent genome-wide studies in patients with Type 2 diabetes.

**Methods:**

We evaluated 16 candidate single nucleotide polymorphisms for estimated GFR in 3028 patients with Type 2 diabetes sampled from clinics across Tayside, Scotland, UK, who were included in the Genetics of Diabetes Audit and Research Tayside (GoDARTs) study. These single nucleotide polymorphisms were tested for their association with estimated GFR at entry to the study, with albuminuria, and with time to stage 3B chronic kidney disease (estimated GFR<45 ml/min/1.73 m^2^). We also stratified the effects on estimated GFR in patients with (*n* = 2096) and without albuminuria (*n* = 613).

**Results:**

rs1260326 in *GCKR* (β=1.30, *P* = 3.23E-03), rs17319721 in *SHROOM3* (β = −1.28, *P*-value = 3.18E-03) and rs12917707 in *UMOD* (β = 2.0, *P*-value = 8.84E-04) were significantly associated with baseline estimated GFR. Analysis of effects on estimated GFR, stratified by albuminuria status, showed that in those without albuminuria (normoalbuminura; *n* = 613), *UMOD* had a significantly stronger effect on estimated GFR (β_normo_ = 4.03 ± 1.23 vs β_albuminuria_ = 1.72 ± 0.76, *P* = 0.002) compared with those with albuminuria, while *GCKR* (β_normo_ = 0.45 ± 0.89 vs β_albuminuria_ = 1.12 ± 0.55, *P* = 0.08) and *SHROOM3* (β_normo_ = −0.07 ± 0.89 vs β_albuminuria_ = −1.43 ± 0.53, *P* = 0.003) had a stronger effect on estimated GFR in those with albuminuria. *UMOD* was also associated with a lower rate of transition to stage 3B chronic kidney disease (hazard ratio = 0.83[0.70, 0.99], *P* = 0.03).

**Conclusion:**

The genetic variants that regulate estimated GFR in the general population tend to have similar effects in patients with Type 2 diabetes and in this latter population, it is important to adjust for albuminuria status while investigating the genetic determinants of renal function.

## Introduction

Recent genome-wide association studies have identified several genetic variants associated with estimated (e)GFR and chronic kidney disease (CKD). Previous investigations of these eGFR polymorphisms were typically carried out in populations where < 10% of patients were diagnosed with Type 2 diabetes 1. It remains to be established if these variants are associated with eGFR in patients with Type 2 diabetes for whom there are different reasons for loss of renal function, in particular diabetic nephropathy, when compared with patients without diabetes. Most of these studies are cross-sectional 2–5, and so clinically relevant dynamic phenotypes cannot be studied. Longitudinal datasets capturing renal function can be used to investigate if the genetic variants identified are associated with a rapid decline in renal function (end-stage renal disease or stage 3 CKD) in patients with Type 2 diabetes. About 20% of patients with Type 2 diabetes with CKD defined according to the ADA guidelines may have normoalbuminuria (albumin/creatinine ratio [ACR] <2.5 mg/mmol in males and ACR<3.5 mg/mmol in females) 6. The genetic and pathological mechanisms that determine the relationship between reduced eGFR and albuminuria status in patients with Type 2 diabetes remain unknown 7. Although the genetic variants associated with eGFR do not seem to be associated with albuminuria 8, it remains to be seen if these genetic variants have the same effect on eGFR in those with and without albuminuria. In the present study, using a longitudinal cohort of patients with Type 2 diabetes, we investigated the association of 16 recently identified eGFR-associated loci (*LASS2, GCKR, NAT8, TFDP2, SHROOM3, DAB2, SLC34A1, VEGFA, PRKAG2, ADAM28, PIP5K1B, ATXN2, DACH1, UBE2Q2, UMOD, SLC7A9*) with baseline eGFR albuminuria, and time to stage 3B CKD (eGFR<45 ml/min/1.73 m^2^), in patients with Type 2 diabetes.

**What’s new?**

- This is the first study comparing common genetic variants associated with estimated GFR between the general population and patients with Type 2 diabetes.
- This is the first report of the interaction of genetic effects of estimated GFR-associated loci (UMOD GCKR and SHROOM3) with albuminuria in patients with Type 2 diabetes.
- The study stresses the need to adjust for albuminuria while investigating the genetic determinants of renal function.

## Methods

The study population comprised 3028 patients with Type 2 diabetes identified from an on-going study, the Genetics of Diabetes Audit and Research Tayside (GoDARTs) study, and recruited in Tayside, Scotland, UK, between 1 October 1997 and 1 March 2010. The baseline clinical characteristics of the GoDARTs subset included in the present analyses were very similar to the baseline clinical characteristics of the remaining GoDARTS cohort, except that those not included were slightly older and had a lower eGFR (Table 1); therefore, the subset of patients used for the present analysis was very representative of the entire GoDARTs cohort**.** Calculations for eGFR were made using the Modification of Diet in Renal Disease formula 9 which requires age, sex, race and creatinine data. We assessed the association of the 16 single nucleotide polymorphisms (SNPs) with eGFR at baseline by linear regression analysis using the gPLINK program 10, adjusting for age, sex, BMI, population structure, HbA_1c_, duration of diabetes and systolic blood pressure. To investigate whether the association of these loci with eGFR differed according to albuminuria status, we carried out a stratified analysis in patients with sustained normoalbuminuria (ACR <2.5 mg/mmol in males and <3.5 mg/mmol in females and with a duration of diabetes >15 years at end of follow-up) and in those with any albuminuria (ACR ≥2.5 mg/mmol in males and ≥3.5 mg/mmol in females, either at baseline or at the end of follow-up).

**Table 1:** Demographic characteristics of the GoDARTs cohort

Characteristic, mean (sd)	GoDARTs cohort in the present study	GoDARTs cohort not included in the present study
Age at baseline, years	59.1 (11.0)	66.2 (11.6)
Sex,% female	46.4	42.3
Baseline BMI	30.6 (5.3)	31.5 (6.1)
Baseline eGFR, ml/min/1.73m^2^	73.9 (18.7)	70.9 (15.8)
Baseline systolic blood pressure, mmHg	142.8 (18.4)	141.7 (18.8)
Baseline HbA_1C_, mmol/mol	7.54 (1.3) (58 mmol/mol)*	7.3 (1.4) (56 mmol/mol)*
Baseline cholesterol, mmol/L	4.40 (0.97)	4.34 (0.91)
Duration of diabetes at baseline, years	8.71 (7.44)	7.75 (6.61)

* These are HbA1c values in IFCC units.

To investigate if any of these SNPs were associated with a rapid decline in renal function over the follow-up period, we performed an analysis of time to stage 3B CKD (eGFR<45 ml/min/1.73 m^2^). Individuals with stage 3B CKD at baseline were excluded. By using this threshold, 4% of our patients were excluded from the analysis. If we had chosen to study progression to stage 3A CKD (eGFR<60 ml/min/1.73 m^2^), 20% of patients would have been excluded from the analysis. Stage 3B CKD was defined as three consecutive eGFR measurements of eGFR <45 ml/min/1.73 m^2^ at least 1 month apart. Those who did not progress to stage 3B CKD were censored at the end of the follow-up period or at date of death. We used a Cox proportional hazards model (the Proc PHREG tool in the sas statistical package), with date of birth as ‘time in’ and ‘last date’ as the first date of eGFR <45 ml/min/1.73 m^2^ or the end of follow-up period/date of death, and with genotype, age, sex, BMI and baseline eGFR as covariates. The interaction of individual SNPs with albuminuria was tested using PLINK option ‘interaction’ with age, sex, BMI, albuminuria and genotypes as covariates in the linear regression model. We adopted a conservative threshold for significance (0.05/number of loci tested) and a *P* value < 0.003 was considered to indicate statistical significance. A weighted genetic risk score analysis was performed to test the joint effect of the 16 loci on baseline eGFR and time to stage 3B CKD. We calculated weighted genetic risk score (number of risk alleles*β) for each individual using all 16 SNPs, and tested the association of this genetic risk score with baseline eGFR and time to stage 3B CKD, adjusting for age, sex, BMI, HbA_1c_, duration of diabetes, and systolic blood pressure. All analyses were performed in PLINK version 1.07^10^ and SAS 9.2. Power calculations for quantitative traits were performed using R 2.15.

Samples were genotyped at Affymetrix’s service laboratory on the Genome-Wide Human SNP Array 6.0. Complete genotype data have been described previously 11. The study complied with the Declaration of Helsinki guidelines. Since October 1997, all individuals with diabetes in the GoDARTs database have been invited to give consent for DNA collection as part of the Wellcome Trust United Kingdom Type 2 Diabetes case–control collection. As of June 2009, 8000 cases and 7000 control subjects of European ancestry have participated in this GoDARTS study. Informed consent was obtained from all the study participants.

## Results

Table 1 shows the baseline characteristics of the GoDARTs cohort included in the present study as well as the GoDARTs cohort not genotyped at the conception of this study. Genotype data were available for 3028 patients (46.4% females) with Type 2 diabetes. Their mean (sd) baseline BMI was 30.6 (5.3) kg/m^2^, mean (sd) age was 59.1 (11) years, mean (sd) HbA_1c_ was 58 mmol/mol (7.54 (±1.3). The mean (sd) follow-up period for the entire study was 10.6 (9.1) years with a median of three eGFR readings/year/person (interquartile range 2–4) and a mean (sd) baseline eGFR of 73.9 (18.7) ml/min/1.73 m^2^.

Table 2 shows the association found for the 16 eGFR-associated loci with baseline eGFR and albuminuria; the study population was stratified by albuminuria status and the association of these SNPs with time to stage 3B CKD. The minor alleles ‘T’ of *GCKR* (β = 1.30, *P*-value = 3.23E-03), and ‘T’ of *UMOD* (β = 2.0 *P*-value = 8.84E-04) were associated with a higher eGFR at baseline and the minor ‘A’ of *SHROOM3* (β = −1.28, *P*-value = 3.18E-03) was associated with a lower eGFR at the predefined threshold (*P* ≤ 0.003). None of the other SNPs was associated with baseline eGFR. None of the 16 SNPs included in the study were associated with albuminuria after correction for multiple testing (data not shown). In patients with sustained normoalbuminuria (*n* = 613), minor allele ‘T’ of *UMOD* was associated with eGFR (β = 4.03, *P*-value = 1.10E-03), while in patients with albuminuria (*n* = 2096) minor allele ‘T’ of *GCKR* (β = 1.12, *P*-value = 4.27E-02) and ‘A’ of *SHROOM3* (β = −1.43, *P*-value = 7.28E-03) were associated with eGFR. Of the 16 SNPs, *UMOD* (hazard ratio = 0.83(0.70, 0.99), *P*-value = 0.03), *PIP5K1B* (hazard ratio = 0.85(0.75, 0.96), *P*-value = 0.01) and *SLC7A9* (hazard ratio = 0.86(0.76, 0.98) *P*-value = 0.02) was associated with time to stage 3B CKD (eGFR<45 mls/min/1.73 m2) at the 0.05 threshold for significance. Although the *PIP5K1B* locus was significant at *P* < 0.05, the direction of effect was not consistent with a previous report by Köttgen *et al*. 4 and hence this cannot be regarded as a positive replication of this SNP for its association with eGFR and time to CKD stage 3B.

**Table 2:** Association of the known single nucleotide polymorphisms with baseline estimated GFR, estimated GFR stratified by albuminuria status and time to stage 3B chronic kidney disease

CHR	Gene	SNP	Effect allele	Association with baseline eGFR (*n* = 2970)		Association with eGFR in patients with sustained normoalbuminuria† (*n* = 613)		Association with eGFR in patients with albuminuria (*n* = 2097)		Interaction term Heterogeneity *P*-value	Association with time to Stage 3B CKD (eGFR<45)*‡		Direction of effect in GoDARTs consistent with Köttgen *et al*. 4
				β (se)	*P*-value	β (SE)	*P*-value	β (se)	*P*-value		Hazard ratio (CI)	*P*-value	
1	LASS2	rs267734	C	0.77 (±0.51)	1.30E-01	2.24 (±1.07)	3.63E-02	0.71 (±0.62)	2.57E-01	9.60E-02	1.12 (0.98,1.29)	7.00E-02	Yes
2	GCKR	rs1260326	T	1.30 (±0.44)	3.23E-03	0.45 (±0.89)	6.12E-01	1.12 (±0.55)	4.27E-02	8.70E-02	0.98 (0.86,1.11)	7.60E-01	Yes
2	NAT8	rs13538	G	0.40 (±0.51)	4.32E-01	0.55 (±1.12)	6.24E-01	0.29 (±0.62)	6.34E-01	8.92E-01	1.02 (1.023,1.027)	2.70E-01	Yes
3	TFDP2	rs347685	C	−0.51 (±0.48)	2.82E-01	0.54 (±0.97)	5.77E-01	−1.07 (±0.59)	6.76E-02	3.95E-01	0.96 (0.83,1.10)	5.50E-01	No
4	SHROOM3	rs17319721	A	−1.28 (±0.43)	3.18E-03	−0.07 (±0.89)	9.34E-01	−1.43 (±0.53)	7.28E-03	3.00E-03	1.02 (0.90,1.15)	6.90E-01	Yes
5	DAB2	rs11959928	A	−0.43 (±0.45)	3.39E-01	−1.45 (±0.90)	1.07E-01	−0.29 (±0.55)	5.99E-01	3.41E-01	0.97 (0.86,1.10)	7.00E-01	Yes
5	SLC34A1	rs6420094	G	−1.35 (±0.61)	2.74E-02	−2.92 (±1.24)	1.87E-02	−0.69 (±0.75)	3.60E-01	2.79E-01	0.93 (0.78.1.10)	4.00E-01	Yes
6	VEGFA	rs881858	G	0.54 (±0.48)	2.63E-01	1.31 (±1.01)	1.92E-01	1.34 (±0.59)	2.21E-02	4.40E-02	0.95 (0.83,1.08)	4.70E-01	Yes
7	PRKAG2	rs7805747	A	−0.31 (±0.49)	5.24E-01	−0.72 (±0.98)	4.62E-01	0.31 (±0.60)	6.02E-01	9.30E-01	1.03 (0.90.1.19)	6.00E-01	Yes
8	ADAM28	rs10109414	T	−0.51 (±0.44)	2.41E-01	−1.57 (±0.90)	8.17E-02	−0.17 (±0.54)	7.49E-01	5.10E-01	0.99 (0.87,1,12)	8.70E-01	Yes
9	PIP5K1B	rs4744712	A	0.09 (±0.44)	8.47E-01	1.71 (±0.91)	6.25E-02	−0.33 (±0.55)	5.41E-01	9.31E-01	0.85 (0.75,0.96)	1.00E-02	No
12	ATXN2	rs653178	T	0.20 (±0.42)	6.28E-01	0.71 (±0.85)	4.05E-01	−0.13 (±0.52)	8.09E-01	9.47E-01	0.95 (0.83,1.08)	9.50E-01	Yes
13	DACH1	rs626277	C	0.75 (±0.44)	9.14E-02	0.85 (±0.90)	3.46E-01	0.28 (±0.54)	6.02E-01	3.93E-01	0.98 (0.87,1.10)	7.50E-01	Yes
15	UBE2Q2	rs1394125	A	−0.86 (±0.53)	1.03E-01	−1.14 (±1.07)	2.89E-01	−0.86 (±0.65)	1.85E-01	2.68E-01	1.11 (0.96,1.28)	1.50E-01	Yes
16	UMOD	rs12917707	T	2.0 (±0.60)	8.84E-04	4.03 (±1.23)	1.10E-03	1.72 (±0.76)	2.30E-02	2.00E-03	0.83 (0.70.0.99)	3.00E-02	Yes
19	SLC7A9	rs12460876	C	0.24 (±0.51)	6.90E-01	0.58 (±0.94)	5.30E-01	0.29 (±0.57)	6.00E-01	4.50E-01	0.86 (0.76,0.98)	2.00E-02	Yes

* Adjusted for age at baseline, duration of diabetes, baseline-estimated GFR, systolic blood pressure, mean HbA_1c_ and mean BMI.

† Patients with normoalbumiuria at baseline and at the end of follow-up with a duration of diabetes >15 years.

‡ Stage 3B CKD defined as three consecutive readings of eGFR <45 ml/min/1.73 m^2^. Those already at stage 3B CKD at baseline were excluded for this analysis.

SNP, single nucleotide polymorphism; CKD, chronic kidney disease; CHR, chromosome.

Since the variants tested in this study are associated with age-related decline in eGFR in general population (and not with any disease-specific decline) we used time-to-event analysis with date of birth as the starting point; however, we performed a sensitivity analysis in which we used the baseline of GoDARTs study as the starting point. Although this analysis decreases power because of a reduction in the person-years follow-up, we see a similar effect size of association with progression to stage 3B CKD. For example, the hazard ratio of *UMOD* with time to stage 3B CKD with the starting point as the GoDARTs study baseline (hazard ratio = 0.87(0.74, 1.03) *P*-value = 0.1) is very similar to the hazard ratio with date of birth as a starting point. The weighted genetic risk score for the 16 SNPS explained the 1% variation in baseline eGFR and was significantly associated with baseline eGFR after adjustments for age, sex, BMI, HbA_1c_, duration of diabetes and systolic blood pressure (*P* = 0.0026, β = 0.84(±0.28). The weighted genetic risk score was not associated with time to stage 3B CKD (*P* = 0.52).

## Discussion

In the present study, we replicated the association of *UMOD, GCKR* and *SHROOM3* with eGFR in patients with Type 2 diabetes. The study confirms the findings of previous studies showing the association of *UMOD* with eGFR and diabetic nephropathy 12–15 and the association of *GCKR* and *SHROOM3* with eGFR 1,16,17. A study by Gudbjartsson *et al*. 12 demonstrated the interaction of *UMOD* with age 15; while another study could not replicate this interaction. In the present study, we did not observe an interaction of *UMOD* with age in patients with Type 2 diabetes (*P*-value = 0.84).

None of the other variants were associated with eGFR after correction for multiple testing; however, the direction of effect was consistent with the previous studies for all the statistically significant loci (*GCKR, SHROOM3, UMOD*) and for the loci that did not pass the threshold of significance (except *TFDP2* and *PIP5K1B*). Our study had limited power to estimate the effect of these variants on eGFR. Taken together, all these variants explain the 1.4–14% heritability of eGFR 5 (with each SNP contributing typically < 0.5% heritability of eGFR). Our study had 97% power to detect an association with a SNP explaining 0.5% variability in eGFR and anything below 0.5% can remain undetected. It is also possible that some of these SNPs are not the causal SNPs and because of varying linkage disequilibrium, structure in our population could not be detected. It is also possible that the effects of some of these SNPs were attenuated by diabetes or diabetic kidney disease and therefore were not associated with eGFR in this study.

We examined the association of the 16 loci with a decline in renal function using a Cox proportional hazard model and estimated the effect of these loci on time to stage 3B CKD (eGFR<45ml/min/1.73 m^2^). Given the high mortality associated with diabetic nephropathy, cross-sectional studies are prone to survival bias, as patients with severe forms of nephropathy are less likely to be included. Hence, it is important to investigate the eGFR loci in a time-dependent manner. Of the 16 SNPs, none were associated with time to stage 3B CKD at the predefined threshold of 0.003, however, *UMOD* and *SLC7A9* were associated with time to stage 3B CKD at the threshold of 0.05 (with the direction of effects consistent with that reported previously). *UMOD* and *SLC7A9* have a stronger effect on baseline eGFR as compared with other markers suggesting that SNPs with a strong effect on baseline eGFR influence the decline in renal function over time.

We performed a stratified analysis to examine the effect of albuminuria on the known genetic associations with eGFR. In Type 2 diabetic, nephropathy, albuminuria may be more closely associated with decline in renal function and the impact of genetic determinants of eGFR may differ depending on the presence or absence of nephropathy; therefore, we examined the effects on eGFR stratified by albuminuria. There is a clear difference in the effect sizes in those with sustained normalbuminuria and those with albuminuria. For example, the *UMOD* has twice the effect in patients with sustained normalbuminuria as compared with those with albuminuria (P-interaction = 0.002) while *SHROOM3* (P-interaction = 0.003) and *GCKR* (P-interaction = 0.08) had larger effect sizes in those with albuminuria. It is known that kidney diseases characterized by albuminuria, such as diabetic nephropathy can have ultrafiltration and high eGFR in the early stage of disease, while those characterized by reduced renal function such as hypertensive kidney disease, may be manifested with normoalbumiuria because of the reduced renal efficiency 18,19. Hence, studying the genetic determinants of eGFR without adjusting for albuminuria status or studying genetic determinants of albuminuria without accounting for eGFR can reduce the power of these studies to identify the true genetic effects. Cumulatively, eGFR-associated loci explain only a small fraction of the total heritable contribution eGFR and stratifying by albuminuria status in our existing genome-wide association study datasets 3–5 can help us to uncover the missing heritability. It is important to point out, however, that the interaction of albuminuria with the genetic variants associated with eGFR in patients with Type 2 diabetes seen in the present study is the first report of this interaction in patients with Type 2 diabetes and needs to be confirmed in an independent sample.

In summary, our results show that some of the genetic determinants of eGFR in the general population are common to patients with Type 2 diabetes; however, in patients with Type 2 diabetes it is essential to adjust for albuminuria status while investigating the genetic determinants of renal function.

### Funding sources

The Wellcome Trust provides support for the Wellcome Trust United Kingdom Type 2 Diabetes Case Control Collection (the Go-DARTS study) and the Chief Scientist Office provides informatics support. The Wellcome Trust funds the Scottish Health Informatics Programme. The Wellcome Trust (084726/Z/08/Z, 085475/Z/08/Z, and 085475/B/08/Z) funded genome-wide genotyping as part of WTCCC2. The IMI SUMMIT programme supported HD.

### Competing interests

None declared.
